# Antimicrobial Activity of Manganese(I) Tricarbonyl Complexes Bearing 1,2,3-Triazole Ligands

**DOI:** 10.3390/molecules28217453

**Published:** 2023-11-06

**Authors:** Sofia Friães, Cândida Trigueiros, Clara S. B. Gomes, Alexandra R. Fernandes, Oscar A. Lenis-Rojas, Marta Martins, Beatriz Royo

**Affiliations:** 1 Instituto de Tecnologia Química e Biológica António Xavier, ITQB NOVA, Avenida da República, 2780-157 Oeiras, Portugal; sofiafriaes@itqb.unl.pt; 2 Department of Microbiology, Moyne Institute of Preventive Medicine, School of Genetics and Microbiology, Trinity College Dublin, The University of Dublin, D02 PN40 Dublin, Ireland; 3 LAQV-REQUIMTE and UCIBIO—Applied Molecular Biosciences Unit, Departamento de Química, Faculdade de Ciências e Tecnologia, Universidade NOVA de Lisboa, 2829-516 Caparica, Portugal; clara.gomes@fct.unl.pt; 4 Associate Laboratory i4HB—Institute for Health and Bioeconomy, NOVA School of Science and Technology, NOVA University Lisbon, 2819-516 Caparica, Portugal; ma.fernandes@fct.unl.pt; 5 UCIBIO—Applied Molecular Biosciences Unit, Department of Life Sciences, NOVA School of Science and Technology, NOVA University Lisbon, 2819-516 Caparica, Portugal

**Keywords:** manganese tricarbonyl complexes, azoles or azole derivatives, antibacterial activity

## Abstract

Background. Antimicrobial resistance is one of the most pressing health issues of our time. The increase in the number of antibiotic-resistant bacteria allied to the lack of new antibiotics has contributed to the current crisis. It has been predicted that if this situation is not dealt with, we will be facing 10 million deaths due to multidrug resistant infections per year by 2050, surpassing cancer-related deaths. This alarming scenario has refocused attention into researching alternative drugs to treat multidrug-resistant infections. Aims. In this study, the antimicrobial activities of four manganese complexes containing 1,2,3,-triazole and clotrimazole ligands have been evaluated. It is known that azole antibiotics coordinated to manganese tricarbonyl complexes display interesting antimicrobial activities against several microbes. In this work, the effect of the introduction of 1,2,3,-triazole-derived ligands in the [Mn(CO)_3_(clotrimazole)] fragment has been investigated against one Gram-positive bacterium and five Gram-negative bacteria. Methods. The initial antimicrobial activity of the above-mentioned complexes was assessed by determining the minimum inhibitory and bactericidal concentrations using the broth microdilution method. Growth curves in the presence and absence of the complexes were performed to determine the effects of these complexes on the growth of the selected bacteria. A possible impact on cellular viability was determined by conducting the MTS assay on human monocytes. Results. Three of the Mn complexes investigated (**4**–**6**) had good antimicrobial activities against all the bacteria tested, with values ranging from 1.79 to 61.95 µM with minimal toxicity. Conclusions. Due to the increased problem of antibiotic resistance and a lack of new antibacterial drugs with no toxicity, these results are exciting and show that these types of complexes can be an avenue to pursue in the future.

## 1. Introduction

Antimicrobial resistance has recently been recognised by the World Health Organization (WHO) as one of the greatest threats to global health. During the last few decades, the number of antibacterial agents that reached the market has decreased and failed to meet the challenges posed to the treatment of multidrug-resistant pathogens [1,2,3]. Therefore, the development of new classes of antimicrobials with alternative modes of action capable to treat these pathogens is urgently needed. Recently, organometallic complexes have emerged as effective agents against pathogenic bacteria and parasitic microorganisms, with potential for clinical development [4,5,6,7]. Organometallic complexes present a metal-specific mode of action that is not available by administering pure organic drugs. In fact, one of the most representative dominant examples in medicinal organometallic chemistry is ferroquine, an effective antimalarial drug [8]. However, in contrast to the significant advances in the development of organometallics as anticancer [9,10,11] and antimalarial drugs [8,12,13,14] the development of organometallic antibacterial agents has received little attention. Only recently a number of organometallic complexes of Au [10,14], Mn [15,16], Ru [17], Rh [18,19], and Ir [18,19,20,21] have been explored as potential antimicrobial drugs. Manganese has recently attracted great interest for biomedical applications, since it is a biocompatible metal playing a critical role in biochemical reactions occurring in human bodies [22]. Moreover, Mn is an essential micronutrient that plays a critical role in the survival and virulence of pathogenic bacteria [23]. This unique feature of Mn can be harnessed for obtaining Mn-based antibacterial complexes [24]. In particular, manganese(I) tricarbonyl complexes containing chelating N-based ligands are appealing as chemical platforms for biological applications [15,25,26,27,28,29]. Interestingly, complexes of the general type [Mn(CO)_3_(bpy)(azole)] (bpy = 2,2’-bipyridine; azole = ketoconazole, miconazole, clotrimazole) have shown promising activity as antibacterial and antiparasitic drugs against *Staphylococcus aureus*, *Staphylococcus epidermidis*, *Leishmania major*, and *Trypanosoma brucei*, and [Mn(CO)_3_(L-L)(clotrimazole)] (L-L = 2,2′-bipyridine and 2,2′-biquinoline) were also active against *Staphylococcus aureus* [15,16]. As shown in these works, the coordination of azole-based ligands to the [Mn(CO)_3_(N-N)] fragment increases the activity of the complexes when compared to purely organic azole agents [15].

Motivated by these results, we decided to investigate the effect of replacing the bipyridyl system in [Mn(CO)_3_(bpy)(clotrimazole)] complexes with 1,2,3-triazole-derived ligands. The 1,2,3-triazole ring is a major pharmacophore system among nitrogen-containing heterocycles [30]. This type of complexes and their 1,2,3-triazole containing hybrids and conjugates have been demonstrated as anticancer [30,31], antimicrobial [32], antiviral [33], antimalarial [34], and anti-tubercular agents [35]. An important feature of 1,2,3-triazole-derived complexes is their easily tuneable synthesis using “click chemistry” with copper- or ruthenium-catalysed azide–alkyne cycloaddition reactions, which allows the direct preparation of a library of complexes [36]. Here, we describe the synthesis and characterisation of Mn(I) tricarbonyl complexes bearing chelating C4-linked di-triazoles and pyridine-derived triazoles of general formula [Mn(CO)_3_(N-N)Br] and [Mn(CO)_3_(N-N)(clotrimazole)]^+^ (N-N = di-triazole, mixed pyridine-triazole) and their potential activities as antimicrobial agents against Gram-positive and -negative bacteria. The potential antimicrobial effects of these complexes were studied against *S. aureus*, *Escherichia coli*, *Salmonella enterica* serovar typhimurium, and *Klebsiella pneumoniae*, since these pathogenic bacteria are leading causes of associated deaths worldwide.

## 2. Results and Discussion

### 2.1. Synthesis

**Scheme 1:**
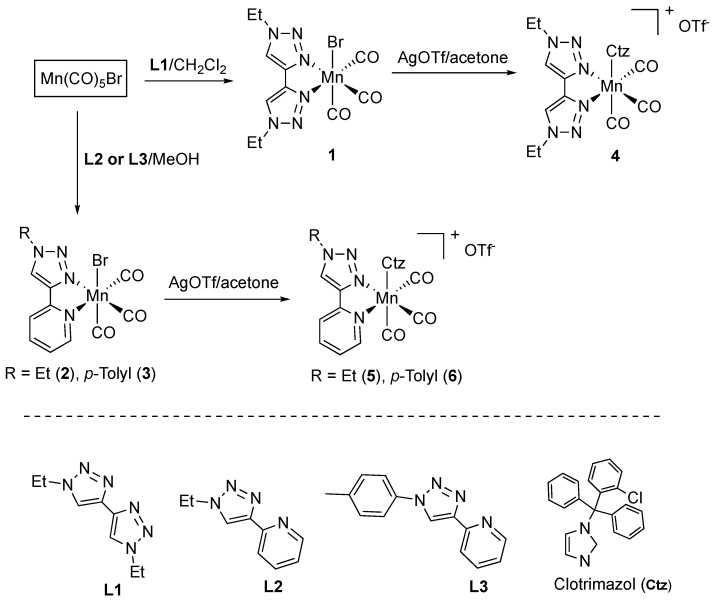
Synthesis of Mn(I) tricarbonyl complexes **1**–**6**.

**Figure 1:**
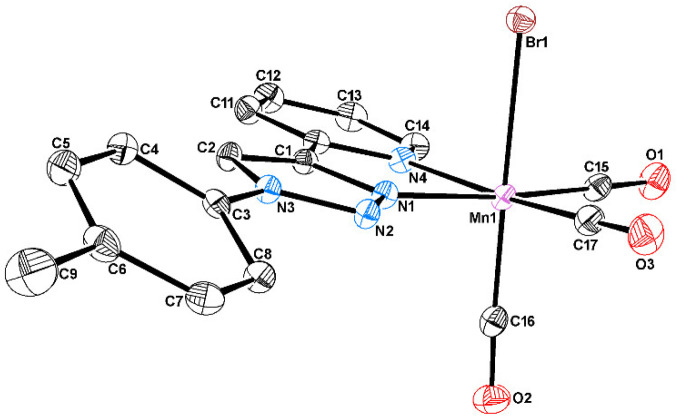
ORTEP representation of complex **3**. Hydrogen atoms and co-crystalised solvent molecules are omitted for clarity. Selected bond lengths: Mn1–Br1 2.5366(3) Å, Mn1–N1 2.0195(16) Å, Mn1–N4 2.0780(16) Å, 1.8075(19) Å, and < Mn1–C_CO_ < 1.812(2) Å. Selected bond angles: N1–Mn1–N4 78.30(6)°, N1–Mn1–C16 173.01(7)°, N4–Mn1–C17 170.99(8)°, and Br1–Mn1–C16 178.98(7)°.

The C4-linked ditriazole **L1** [37] and the pyridine-derived triazoles **L2** [38] and **L3** [36] were prepared following the well-established copper-catalysed click [3+2] cycloaddition reaction previously described in the literature. Complexes **1** and **2** have been already reported by us in [39]. They were prepared via the direct reaction of MnBr(CO)_5_ with the appropriate **L1** and **L2** ligands, as depicted in Scheme 1. Following the same procedure, complex **3** was prepared and purely isolated. The treatment of complexes **1**–**3** with one equivalent of AgOTf for bromide abstraction and subsequent addition of clotrimazole (Ctz) yielded the formation of the cationic species *fac*-[Mn(CO)_3_(L)(Ctr)][OTf] (L = **L1** (**4**), **L2** (**5**), **L3** (**6**)), which were isolated as air stable, crystalline solids after recrystallisation in a mixture of dichloromethane/ether (1:4). The identities and purities of all new complexes have been established via NMR and IR spectroscopy, elemental analysis, and, in the case of **3**, X-ray diffraction studies (Figure 1). Upon the complexation of **L1**–**L3** to the tricarbonyl manganese core, significant downfield shifts in the resonance of the triazole and pyridine protons were observed. The chelation of the pyridine unit in complexes **2** and **3** is supported by the low-field resonance of the proton in the pyridine ortho position from δ_H_ 8.6 to 9.2 (for **2**) and δ_H_ 8.6 to 9.8 (for **3**). The IR spectra of complexes **1**–**6** showed the characteristic pattern of tricarbonyl species in a facial arrangement of the CO ligands, displaying three intense stretching bands in the 2034–1917 cm^−1^ region. As expected, the cationic species **4**–**6** displayed the symmetric CO stretching frequency at higher values than those shown by the corresponding neutral complexes (e.g., 2043 (for **4**) vs. 2034 (for **1**) cm^−1^) [40].

Complex **3** crystallised as yellow needles in the monoclinic system, space group *P*2_1_/*c*, displaying one molecule in the asymmetric unit. The Mn(I) metal center displays a slightly distorted octahedral geometry, with all bond angles being very close to the expected 90°, Figure 1. The Mn–N bond distances to the N,N’– *p*-tolyl-triazolyl-pyridine chelating ligand are 2.0195(16) for Mn1–N1 and 2.0780(16) Å for Mn1–N4, being smaller than those reported for compound [Mn(N-N)_2_Cl] [41]. The Mn1–Br1 bond length is 2.5366(3), whereas the Mn–C_CO_ bond distances vary between 1.8075(19) and 1.812(2) Å. The N,N’– *p*-tolyl-triazolyl-pyridine chelating ligand displays an N1–Mn–N4 bite angle of 78.30(6)°. The dihedral angle between the triazolyl and pyridine rings is 1.70(7)°, reflecting the essentially planar backbone of the ligand. This is due to the chelation effect to the Mn(I) metal center that forces the ligand into a planar conformation. On the other hand, the angle between the planes containing the *p*-tolyl and the triazolyl rings is 21.74(7)°. All the remaining bond distances and angles within the chelating ligand are within the reported values for similar complexes [42].

### 2.2. Antimicrobial Activity of the Complexes against Gram-Positive and -Negative Bacteria

The antimicrobial activity of the cationic complexes **4**–**6** containing the clotrimazole molecule and the neutral complex **1** without clotrimazole were investigated on the bacterial growth of Gram-positive (*S. aureus*) and -negative bacteria. It is known that due to their intrinsic physiological properties, such as the composition of the cell wall, Gram-negative bacteria are more difficult to treat than Gram-positive [43]. Complexes **1** and **4**–**6** were tested against several Gram-negative bacteria usually involved in infection, namely *E. coli*, *S.* typhimurium, *K. pneumoniae*, *P. aeruginosa*, and *A. baumannii*. These bacteria are also part of the groups of pathogens named ESKAPE [7,16,43], which constitute a priority for the development of new drugs. The activity was initially assessed via the broth microdilution method. The results are summarised in Table 1.

The range of minimum inhibitory and bactericidal concentrations (MIC/MBCs) against the selected bacteria were diverse, varying from 1.79 to 1411.13 µM. Compound **1** was the least active against all the bacteria tested, with MIC values of 2822.11 µM (maximum concentration tested), indicating that the presence of the clotrimazole is crucial for the activity of the complexes.

Complexes **4**–**6**, bearing di-triazole ligands (complex **4**) and mixed triazole–pyridine rings (complexes **5** and **6**) with N-ethyl (**5**) and N-tolyl (**6**) substituents showed comparable activity against *S. aureus* with MIC values of 1.89 µM for **4**, 1.93 µM for **5**, and 1.79 µM for **6**. These were also the values for the MBC, showing that all **4**–**6** complexes have bactericidal activity at the same concentrations. Interestingly, when tested against Gram-negative bacteria, complexes **4**–**6** demonstrated good antibacterial activity at MIC concentrations ranging from 1.79 to 61.95 µM. These three complexes showed good activity against *E. coli*, *P. aeruginosa*, and, in particular, *A. baumannii*. These results are very encouraging because, as mentioned above, Gram-negative bacteria are more difficult to treat than Gram-positive bacteria [43], but, importantly, *E. coli*, *P. aeruginosa*, and *A. baumannii* strains are among the six leading pathogens that cause deaths associated with resistance [44]. Complexes **4**–**6** were also active against *Salmonella* and *Klebsiella* at MIC concentrations ranging from 14.38 to 61.95 µM, while the MBC ranged between 28.76 and 247.80 µM.

**Figure 2:**
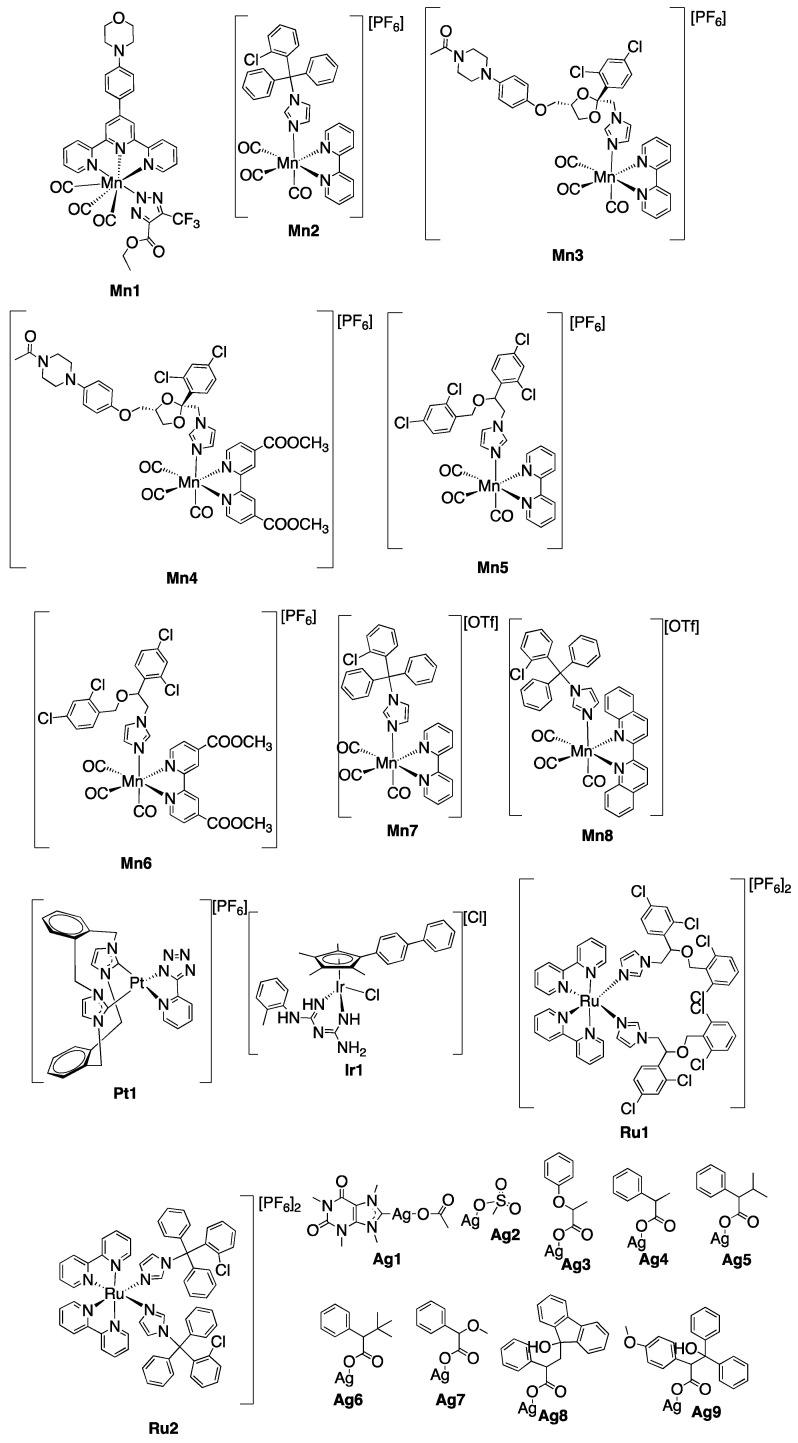
Selected metal complexes reported in the literature as antimicrobial drugs.

Replacing the bipyridyl system in [Mn(CO)_3_(bpy)(clotrimazole)]^+^ complexes with 1,2,3,-triazole-derived ligands resulted in an increase in the antimicrobial activity, in particular against Gram-negative bacteria, in comparison to other metal complexes previously reported in the literature (Figure 2 and Table 2) [7,15,16]. Indeed, complexes **4**–**6** displayed higher activity than **Mn3**, **Mn6**, **Mn7**, and **Mn8** (Figure 2) in *S. aureus*; comparable activity to **Mn5**; and less active to **Mn2** [15,16]. Additionally, the MIC values obtained for **4**–**6** against *E. coli* are higher than those reported for **Mn2**–**Mn8**.

Given the higher risk to public health caused by Gram-negative bacteria, a vast study of the antimicrobial activity of a large number of metal complexes (906 examples) was recently reported, representing only 1.5% of all submitted complexes, as active against Gram-negative strains tested [7]. Gratifyingly, Complexes **4**–**6** were more active than the highest active metal complexes reported in the referred study [7]. In total, 30 of these 906 complexes were selected because they were active and non-toxic. Most of these 30 metal complexes were more active against Gram-positive bacteria than against tested Gram-negative bacteria, such as *Acinetobacter baumannii*, *Escherichia coli*, *Klebsiella pneumoniae*, and *Pseudomonas aeruginosa*. The MIC values reported in the literature for several Ag, Ir, Ru, Pt, and Mn complexes are shown in Table 2. In comparison to these values, complexes **4**–**6**, in most cases, showed significant activity against *E. coli*, *Salmonella typhimurium*, *Klebsiella pneumoniae*, *Pseudomonas aeruginosa*, and *Acinetobacter baumannii*, showing their excellent potential as therapeutic alternatives to Gram-negative bacteria infections.

### 2.3. Effects of the Compound ***4*** on the Bacterial Growth of Gram-Positive and Gram-Negative Bacteria

**Figure 3:**
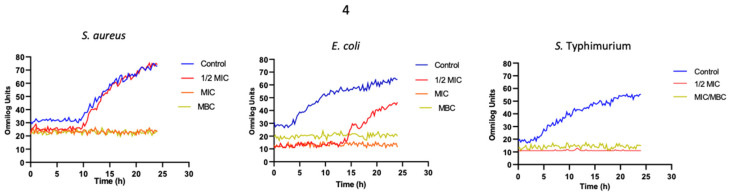
Growth kinetics of *S. aureus* ATCC25923, *E. coli* ATCC 25922, and *Salmonella typhimurium* 14028S in the presence of **4**. Effects of the complexes on the growth of selected bacteria when these were cultured in MH broth. The results correspond to the average of three independent experiments ± standard deviation (SD).

The effects of MIC and sub-MICs of **4** on the growth of the bacteria were monitored in rich media, namely MH broth, in the presence of the MIC and sub-MICs (½ MIC, ¼ MIC, and ^1/8^ MIC) of the complexes. The monitoring of the bacterial growth was performed for 24 h by incubating 96-well plates using a R. Biolog’s OmniLog^®^ PM Sytem automated platform. Untreated controls were prepared in parallel and under growth monitored under the same conditions for each of the strains. At ½ MIC, there was an extension of the lag phase on all the bacteria tested. In the case of the Gram-negative bacteria after the extension of the lag phase, the bacteria were able to grow but never reached the same level as the control. The opposite was obtained with *S. aureus*, which, after the extension of the lag phase, was able to reach the same level of growth as that of the control (no compounds). This extension of the bacterial lag phase was not unexpected, since it is known that bacteria require time to adapt to the presence of toxic compounds. Some examples are given in Figure 3 regarding the growth of *S. aureus*, *E. coli*, and *Salmonella* in the presence of **4**.

### 2.4. Effects of Complexes ***4***–***6*** on the Cellular Viability of Human Monocytes

**Figure 4:**
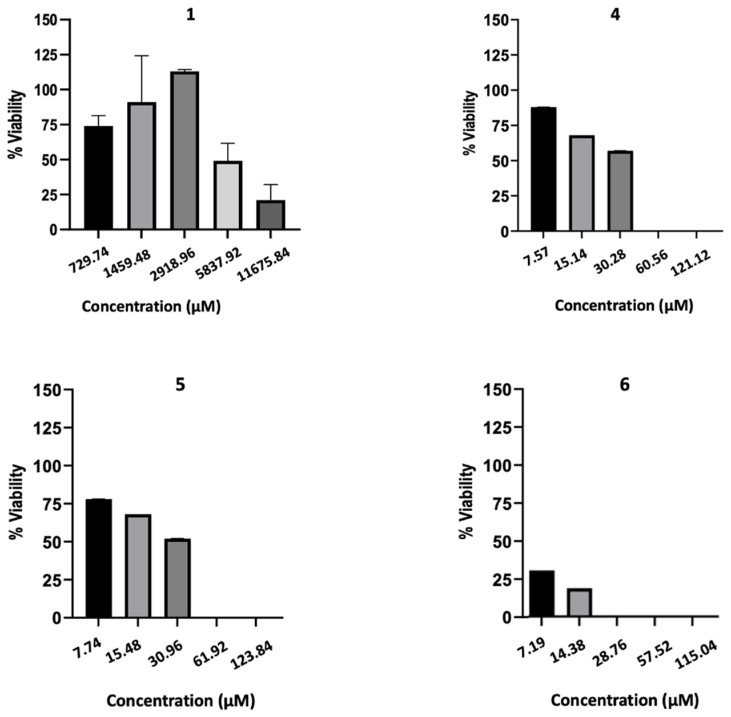
Effect of the complexes on the cellular viability of human monocytes (THP-1 cell line). Human monocytes were exposed to the complexes for 24 h at 37 °C with 5% CO_2_. The assay was performed for all the complexes using concentrations ranging from ¼ MBC to 4× MBC for each compound. The results presented correspond to the average of two independent experiments ± standard deviation (SD); the percentage was compared to controls from two independent biological replicates.

Due to the promising in vitro antimicrobial activity of complexes **4**–**6** against Gram-positive and -negative bacteria, we additionally assessed their possible effect on the cell viability of human monocytes (THP-1 cell line) by conducting an MTS assay. After 24 h of exposure, the viability of the cell line was assessed using the Cell Titer 96^®^AQueous One solution. In brief, the conversion of MTS into an aqueous formazan product is accomplished by dehydrogenase enzymes found in metabolically active cells. As shown in Figure 4, compound **1** had an impact on viability at concentrations of 5645.33 µM or higher. At a concentration of 2822.66 µM, the compound seemed to promote proliferation (due to an increase in viability; viability > 100%), but this will still need to be confirmed via additional tests. At concentrations of 7.57 and 7.74 µM of complexes **4** and **5**, respectively, the human monocytes cells showed 75% (or higher) viability. Concentrations higher than those had an effect on the cell viability for both complexes. Compound **6** at a concentration as low as 7.19 µM had a big impact on the viability of the cells (viability lower than 50%). Overall, the majority of the compounds (**1**, **4**, **5**) had no major adverse impacts on the viability of the cells at the in vitro concentrations of 15.17, 7.57, and 7.74 µM, which is promising.

## 3. Materials and Methods

### 3.1. Synthesis

All syntheses were carried out under nitrogen atmosphere using Schlenk techniques. Solvents were dried prior to use via standard methods. Ligands **L1**–**L3** and complexes **1** and **2** were prepared according to the literature’s procedures [37,38,39]. All other reagents were purchased from commercial suppliers and used without further purification. ^1^H and ^13^C NMR spectra were recorded via a Bruker Avance III at 400 and 500 MHz. IR spectra were recorded as Nujol mulls, polyethylene disk Nujol mulls, or KBr disks on a Satellite FTIR instrument.

#### Synthetic Procedures

**Preparation of 3**. MnBr(CO)_5_ (117 mg, 0.42 mmol) and **L3** (100 mg, 0.42 mol) were dissolved in CH_2_Cl_2_ (15 mL) and refluxed for 16 h. After cooling to room temperature, the solvent was removed under vacuum, and the remaining solid was washed several times with ether to yield complex **3** as a yellow solid (109 mg and 56%). ^1^H NMR (400 MHz, DMSO-d_6_, 298 K) δ: 9.84 (s, 1H, H_trz_), 9.14 (d, *J* = 5.21 Hz, ^1^H, H_py_), 8.24 (t, *J* = 7.50 Hz, 1H, H_py_), 8.17 (d, *J* = 7.61 Hz, 1H, H_py_), 7.90 (d, *J* = 8.21 Hz, 2H, CH_p-tolyl_), 7.66 (t, *J* = 6.31 Hz, 1H, H_py_), 7.52 (d, *J* = 8.13 Hz, 2H, CH_p-tolyl_), and 2.44 (s, 3H, CH_3p-tolyl_). ^13^C NMR (100 MHz, DMSO-d_6_, 298 K) δ/ppm: 223.46 (CO), 222.35 (CO), 221.47 (CO), 154.21, 149.05, 148.08, 140.54, 133.98, 131.07, 130.68, 126.17, 123.36, 122.44, 120.87, and 21.14 (CH_3*p-tolyl*_). IR (KBr pellets): 2030 (vs), 1955 (vs), 1922 (vs) cm^−1^. Elemental Analysis. Calcd. for C_17_H_12_BrMnN_4_O_3_: % C, 44.86; H, 2.66; N, 12.31. Found: C, 44.42; H, 2.69; N, 11.85.

**A general procedure for the synthesis of [Mn(L)(CO)_3_(ctz)]OTf complexes 4**–**6:** Silver triflate (1.2 eq.) was added to a solution of Mn(L)(CO)_3_Br (1 eq.) in degassed acetone (10 mL). The mixture was stirred at ambient temperature in the dark for 3 h. The solution was then filtered, and clotrimazole (1.2 eq.) was added to the filtrate. After stirring in the dark for 20 h, the solvent was removed under vacuum, and the residue was recrystallised via diffusion with a mixture of CH_2_Cl_2_/Ether. The resulting precipitate was collected and dried under a vacuum.

**Complex 4.** Yield: 48%. ^1^H-NMR (400 MHz, DMSO-d_6_, 298 K) δ/ppm: 8.86 (s, 2H), 7.49 (s, 1H), 7.41 (s, 8H), 7.10 (d, *J* = 7.8 Hz, 1H), 7.02 (s, 1H), 6.82 (d, *J* = 7.3 Hz, 3H), 6.77 (d, *J* = 5.2 Hz, 2H), 6.73 (d, *J* = 8.0 Hz, 1H), 4.56 (q, *J* = 7.1 Hz, 4H), and 1.43 (t, *J* = 6.7 Hz, 6H). ^13^C NMR (100 MHz, DMSO-d_6_, 298 K) δ/ppm: 220.26 (CO), 217.38 (CO), 139.70, 139.13, 139.02, 138.63, 137.08, 134.20, 131.98, 130.96, 130.58, 130.37, 139.45, 128.92, 128.49, 128.26, 128.03, 127.41, 123.54, 122.76, 120.83, 75.54, 47.03 (NCH_2_CH_3_), 44.55 (NCH_2_CH_3_), 15.18 (NCH_2_CH_3_), and 14.80 (NCH_2_CH_3_). IR (KBr pellets): 2043 (vs), 1936 (vs). Elemental Analysis Calcd. for C_34_H_29_ClF_3_MnN_8_O_6_S.0.05C_4_H_10_O: % C, 49.56; H, 3.59; N, 13.52; S, 3.87. Found: C, 50.02; H, 3.13; N, 13.41; S,4.21.

**Complex 5.** Yield: 35%. ^1^H-NMR (400 MHz, DMSO-d_6_, 298 K) δ/ppm: 9.23 (s, 1H), 9.16 (d, *J* = 5.2 Hz, 1H), 8.23 (t, *J* = 7.6 Hz, 1H), 8.11 (d, *J* = 7.7 Hz, 1 H), 7.63 (t, *J* = 6.6 Hz, 1H), 7.48 (d, *J* = 8.4 Hz, 1H), 7.44 (d, *J* = 6.4 Hz, 1H), 7.41–7.34 (m, 8H), 7.03 (s, 1H), 6.93 (s, 1H), 6.80 (d, *J* = 8.1 Hz, 2H), 6.76 (d, *J* = 6.2 Hz, 2H), 6.73–6.70 (m, 1H), 4.55 (q, *J* = 7.2 Hz 2H), and 1.43 (t, *J* = 7.3 Hz, 3H). ^13^C NMR (100 MHz, DMSO-d_6_, 298 K) δ/ppm: 220.53 (CO), 218.76 (CO), 217.54 (CO), 154.05, 148.19, 146.86, 140.62, 140.19, 139.62, 139.42, 138.91, 137.85, 134.44, 134.16, 131.99, 130.91, 130.63, 130.39, 130.19, 129.47, 129.04, 128.83, 128.26, 128.05, 127.44, 126.35, 124.95, 123.55, 122.56, 122.13, 75.54, 47.12 (NCH_2_CH_3_), and 14.57 (NCH_2_CH_3_). IR (KBr pellets): 2041 (vs), 1934 (vs). Elemental Analysis. Calcd. for C_35_H_27_ClF_3_MnN_6_O_6_S.0.65CH_2_Cl_2_: % C, 49.66; H, 3.31; N, 9.75; S, 3.72. Found: C, 50.15; H, 2.82; N, 9.31; S, 3.51.

**Complex 6.** Yield: 27% ^1^H-NMR (400 MHz, DMSO-d_6_, 298 K) δ/ppm: 9.87 (s, 1H), 9.24 (d, *J* = 5.2 Hz, 1H), 8.30 (t, *J* = 7.5 Hz, 1H), 8.11 (d, *J* = 7.8 Hz, 1H), 7.73 (d, *J* = 8.4 Hz, 2H), 7.55 (d, *J* = 8.4 Hz, 2H), 7.41 (s, 2H), 7.33–7.31 (m, 8H), 7.04 (s, 1H), 6.99 (s, 1H), 6.87 (s, 1H), 6.81–6.75 (m, 4H), 6.72 (d, *J* = 7.6 Hz, 1H), and 2.47 (s, 3H). ^13^C NMR (100 MHz, DMSO-d_6_, 298 K) δ/ppm: 220.23 (CO), 218.73 (CO), 217.25 (CO), 154.17, 149.20, 147.83, 147.65, 140.67, 140.39, 139.99, 139.72, 139.29, 138.82, 138.30, 137.68, 137.21, 134.36, 134.09, 134.01, 132.99, 131.96, 130.36, 130.01, 129.36, 128.90, 128.74, 128.03, 127.38, 126.54, 123.52, 123.27, 122.09, 121.81, 120.93, 120.31, 119.86, 75.45, and 20.36 (CH_3*p-tolyl*_). IR (KBr pellets): 2041 (vs), 1936 (vs). Elemental Analysis. Calcd. for C_40_H_29_ClF_3_MnN_6_O_6_S: % C, 55.28; H, 3.36; N, 9.67; S, 3.69. Found: C, 54.89; H, 3.20; N, 9.28; S, 4.01.

### 3.2. Crystallography

Crystals suitable for single-crystal X-ray analysis of compound **3** were selected and covered with Fomblin (polyfluoro ether oil) and mounted on a nylon loop. The data were collected at room temperature via a Bruker D8 Venture diffractometer equipped with a Photon 100 CMOS detector using graphite monochromated Mo-Kα radiation (λ = 0.71073 Å). The data were processed using the APEX3 suite software package, which includes integration and scaling (SAINT), absorption corrections [45] (SADABS), and space-group determination (XPREP). The structure solution and refinement were performed using direct methods using the programs SHELXT 2014/5 and SHELXL (version 2018/3) [46,47] via inbuilt APEX and WinGX-Version 2020.1 [48] software packages. All non-hydrogen atoms were anisotropically refined. All hydrogen atoms were inserted in idealised positions and allowed to refine riding on the parent carbon or oxygen atom with C–H distances of 0.93 Å and 0.96 Å for aromatic and methyl H atoms, respectively. The molecular diagrams were drawn using ORTEP3 (version 2020.1) [48] in the software package. Crystal data for **3** were as follows: C_17_H_12_BrMnN_4_O_3_, FW = 455.16, monoclinic, space group *P*2_1_/*c* (no. 14), D*c* = 1.735 g cm^−3^, Z = 4, a = 19.3095(10), b = 13.5001(7), c = 6.6858(3) Å, α = 90, β = 90.145(2), γ = 90 °, V = 1742.85(15) Å^3^, T = 110(2) K. For a Bruker Venture diffractometer with a Photon 100 area detector, λ (MoK_α_) = 0.71073 Å and µ = 3.075 mm^−1^. Of the 40,065 reflections measured, 6684 were unique. Refinement on *F^2^* concluded with the values *R_1_* = 0.0361 and *wR_2_* = 0.0817 for 235 parameters and 5394 data with I > 2σI. The data were deposited in CCDC under the deposit number 2298511.

### 3.3. Biological Studies

#### 3.3.1. Antimicrobial Activity

Bacterial Strains: *Staphylococcus aureus* ATCC25923, *Escherichia coli* ATCC25922, *Salmonella typhimurium* ATCC14028S, *Klebsiella pneumoniae* ATCC70063, *Pseudomonas aeruginosa* PAO1, and *Acinetobacter baumannii* ATCC 19606 were used in this study. For overnight cultures, three colonies isolated from a Mueller–Hinton (MH) streaked agar plate were inoculated into 5 mL of MH broth and cultures incubated at 37 °C with shaking. All bacterial stocks were stored at −80 °C. For the incubation, the Nuaire CO_2_ incubator, IE was used.

The antimicrobial activity of the compounds against Gram-positive and -negative bacteria: The minimum inhibitory concentration (MIC) was determined using the broth microdilution method in a 96-well plate according to the Clinical Laboratory Standards Institute (CLSI) guidelines [49] In brief, overnight cultures were diluted in sterilised phosphate buffer saline (PBS) solution to ~105 colony-forming units (CFU)/mL. Aliquots of 10 µL were then transferred to separate wells in a 96-well plate that contained 100 µL of each compound at varying concentrations in Mueller–Hinton (MH) broth. After incubation at 37 °C for 18 h, the MIC was determined as the lowest concentration of the compound where no visible growth of the bacteria was obtained, i.e., the lowest concentration of compound that was able to ‘visibly’ inhibit the growth of the bacterial culture. Readings were conducted via eyeball inspection. The determination of the minimum bactericidal concentration (MBC) was performed via the replica transfer of the MIC plate into a new 96-well plate with compound-free media. Plates were incubated at 37 °C, and the MBC results were recorded after 18 h. The assays were performed with three biological replicates on three independent days.

The effects of the compounds on the growth of Gram-positive and -negative bacteria: Firstly, 96-well plates were prepared with different concentrations of the compounds in Mueller–Hinton (MH) broth. Diluted overnights cultures of the bacteria (~105 CFU/mL) were added to each well, except to the control wells (sterility control; only media). The microplate was then incubated in a Biolog OmniLog^®^ System (Biolog; Technopath; US), and the bacterial growth was monitored over a period of 24 h at 37 °C. The assays were performed with three biological replicates in three independent days.

The stocks of the compounds were freshly prepared on the day using DMSO. No precipitation or visible cloudiness was seen. The stocks of the compounds were kept in aliquots stored at −20 °C. The dilutions of the compounds were made in the culture media (Mueller–Hinton) on the day of the experiments. DMSO controls were used internally for the determination of any experimental work to rule out any effect of the solvent on the growth or inhibition of the bacterial strains.

#### 3.3.2. Effects of the Compounds on the Viability of Human Monocytes—MTS Assay

The human monocytic cell line THP-1 was purchased from the American Type Culture Collection (ATCC) (www.atcc.org) and cultured according to the manufacturer′s specifications. These cells were grown in suspension in Roswell Park Memorial Institute (RPMI)-1640 media containing Glutamax and supplemented with 10% (*v*/*v*) of FBS in a humidified incubator at 37 °C with 5% CO_2_. THP-1 cells were cultured in 96-well plates (Corning^®^ Costar ^®^, New York, NY, USA) at a density of 2.5 × 105 cells per mL. Cells were then incubated with the compounds for 24 h at 37 °C with 5% CO_2_. Viability assays were performed using the CellTiter 96TM Aqueous Non-Radioactive Cell Proliferation Assay (Promega, Madison, WI, USA) according to the manufacturer’s instructions. In brief, 3-(4,5-dimethylthiazol-2-yl)-5-(3-carboxymethoxyphenyl)-2-(4-sulfophenyl)-2H-tetrazolium, inner salt (MTS)/phenazine metasulfate (PMS) was added to each well, and the cells were incubated for 1 h at 37 °C. Following incubation, the absorbance measurements of the soluble formazan product (brown) were measured via a microplate reader (Synergy™ HT multimode microplate) at 490 nm. The formazan product was directly proportional to the number of living cells. The assays were performed with three biological replicates, unless otherwise stated.

#### 3.3.3. Data Analysis

Data were analysed using Microsoft Excel version 16.66.1 or Prism Graphpad software version 8.0.2.

## 4. Conclusions

The escalating numbers of antibiotic-resistant bacteria worldwide raise the urgency of discovering or synthesising novel classes of antimicrobial complexes. Treatment options that rely on existing antibiotics are becoming less effective; therefore, the reporting of novel complexes with antimicrobial activity is of extreme importance. Here, we demonstrate that manganese complexes have broad-spectrum antimicrobial activity in vitro, but especially against the Gram-positive bacteria *S. aureus*. These complexes were tested for their potential effects on the growth kinetics, as well as a possible impact on the viability of human monocytes. In vitro, complexes **4**–**6** demonstrated good antibacterial activity at MIC concentrations ranging from 1.79 to 61.95 µM. This activity was accomplished against all the Gram-negative bacteria tested, but especially against *E. coli*, *P. aeruginosa*, and *A. baumannii*. These three complexes were also active against *Salmonella* and *Klebsiella* at MIC concentrations ranging from 14.38 to 61.95 µM. Moreover, when tested against human monocytes, compounds **4** and **5** had no negative impacts on the viability of these cells at the concentrations tested in vitro. Complex **6**, however, had a very adverse effect on the viability of human monocytes. Most of the WHO list is made of Gram-negative bacterial pathogens, mainly because Gram-negative bacteria are more resistant to antibiotics than Gram-positive bacteria. Furthermore, no new antibiotic classes have been approved for treating Gram-negative pathogens in the last few decades. Consequently, this scenario represents one of the most serious threats to human health nowadays, and those results are promising in terms of considering these complexes as a possible future alternative to the available arsenal of antibacterial agents.

## Figures and Tables

**Table 1:** MIC and MBC were determined via broth microdilution method, as per Clinical Laboratory Standards Institute (CLSI) guidelines. MIC—minimum inhibitory concentration; MBC—minimum bactericidal concentration. When MIC and MBC values are the same, only one value is presented for the compounds. When MBC values differ from those of the MIC, MBC values are presented within brackets. Tetracycline (antibiotic) and thioridazine (phenothiazine with demonstrated antimicrobial activity) were included as internal controls and for the comparison of activity.

Compounds	Bacterial Strains/MIC and (MBC) (µM)					
	Gram-Positive Bacteria	Gram-Negative Bacteria				
	*S. aureus* ATCC25923	*E. coli* ATCC25922	*S. typhimurium* 14028S	*K. pneumoniae* ATCC700603	*P. aeruginosa* PAO1	*A. baumannii* ATCC19606
**1**	1411.33 (2822.66)	2822.11	2822.11	2822.11	2822.11	2822.11
**4**	1.89	15.15 (30.29)	30.29	60.59	15.15	1.89 (3.79)
**5**	1.93	15.48 (30.98)	30.97	61.95 (247.80)	15.48	1.93 (3.88)
**6**	1.79	7.19 (14.38)	14.38 (28.76)	57.52	7.19 (14.38)	1.79
Tetracycline	0.81	3.51	13.78	56.25 (112.50)	28.12	0.81 (1.75)
Thioridazine	33.73	67.46 (134.93)	539.70	1079.42	269.85	33.73

**Table 2:** MIC (µM) of representative metal complexes with reported antimicrobial activity [7,15,16].

Compounds	Bacterial Strains/MIC (µM)					
	Gram-Positive Bacteria	Gram-Negative Bacteria				
	*S. aureus*	*E. coli*	*S. typhimurium*	*K. pneumoniae*	*P. aeruginosa*	*A. baumannii*
**4**	1.89	15.15	30.29	60.59	15.15	1.89
**5**	1.93	15.48	30.97	61.95	15.48	1.93
**6**	1.79	7.19	14.38	57.52	7.19	1.79
**Mn1**	>43.15	10.78	-	>43.15	>43.15	>43.15
**Mn2**	0.625	40	-	-	> 40	-
**Mn3**	2.5	>40	-	-	> 40	-
**Mn4**	40	>40	-	-	> 40	-
**Mn5**	1.25	20	-	-	> 40	-
**Mn6**	2.5	>40	-	-	> 40	-
**Mn7**	2.53	40.55	-	-	-	-
**Mn8**	8.99	35.98	-	-	-	-
**Pt1**	19.30	38.62	-	38.62	>38.62	>38.62
**Ir1**	1.37	5.50	-	44.03	>44.03	5.50
**Ru1**	0.65	20.83	-	>20.83	>20.83	>20.83
**Ru2**	1.43	22.97	-	>22.97	>22.97	>22.97
**Ag1**	85.07	42.54	-	85.07	42.54	42.54
**Ag2**	20	10	-	20	>20	5.0
**Ag3**	20	20	-	20	>20	5.0
**Ag4**	>20	20	-	>20	>20	20
**Ag5**	>20	>20	-	>20	>20	10
**Ag6**	>20	20	-	>20	>20	10
**Ag7**	>20	20	-	>20	>20	10
**Ag8**	20	20	-	>20	>20	10
**Ag9**	>20	>20	-	>20	>20	20
**Colistin**	-	0.11	-	2.16	2.16	2.16

(-) not determined.

## Data Availability

Data is contained within the article.
